# Survival analysis of age-related oral squamous cell carcinoma: a population study based on SEER

**DOI:** 10.1186/s40001-023-01345-7

**Published:** 2023-10-10

**Authors:** Jingjing Yang, Kaibo Guo, Anlai Zhang, Ying Zhu, Wendi Li, Jieru Yu, Peipei Wang

**Affiliations:** 1 Hangzhou Cancer Hospital, Hangzhou, 310000 ZJ China; 2 Department of Oncology, Affilited Hangzhou First People’s Hospital, Zhejiang University School of Medicine, Hangzhou, 310006 ZJ China; 3 The First Affiliated Hospital of Zhejiang Chinese Medical University (Zhejiang Provincial Hospital of Chinese Medicine), Hangzhou, 310053 ZJ China; 4 School of Basic Medical Sciences, Zhejiang Chinese Medical University, No.548 Binwen Road, Binjiang District, Hangzhou, 310053 ZJ China; 5 Zhejiang Cancer Hospital, Hangzhou Institute of Medicine (HIM), Chinese Academy of Sciences, No.1 Banshan East Road, Gongshu District, Hangzhou, 310022 ZJ China; 6 Dongyang traditional Chinese medicine hospital, No.999 Jiaoshou Road, 322100 Dongyang, China

**Keywords:** Oral squamous cell carcinoma, Age, SEER database, Survival analysis

## Abstract

**Background:**

This research aimed to investigate the prognostic factors of oral squamous cell carcinoma (OSCC), especially the role of age.

**Methods:**

A total of 33,619 cases of OSCC were received from the Surveillance, Epidemiology, and End Results (SEER) database during 2005–2015. Kaplan–Meier curves of 5-year overall survival rates and 5-year cancer-specific survival rates were performed, and univariate and multivariate Cox regression analyses as well as competing risk model were used to help understand the relationship between various factors and mortality of OSCC.

**Results:**

Compared to 18–39-year-old group, the older age was an important predictor of worse prognosis. The multivariate analysis of overall survival (OS) was 50–59 years (HR, 1.32; 95% CI 1.17–1.48; *p* ≤ .001), 60–69 years (HR, 1.66; 95% CI 1.42–1.87; *p* ≤ .001) and 70 + years (HR, 3.21; 95% CI 2.86–3.62; *p* ≤ .001), respectively, while the specific value of competing risk model was 60–69 years (HR, 1.21; 95% CI 1.07–1.38; *p* = .002) and 70 + years (HR, 1.85; 95% CI 1.63–2.10; *p* ≤ .001). In addition, female gender, unmarried, Blacks, tumor in floor of mouth, size and higher Tumor Node Metastasis (TNM) classification were also other predictors that signify significant clinically deterioration of OS/cancer-specific survival (CSS).

**Conclusions:**

Our research revealed that age was an important factor in explaining the difference of survival in the whole process of OSCC. It is suggested that we should pay attention to the influence of age on diagnosis, treatment and prognosis in the clinical process.

## Introduction

Oral squamous cell carcinoma (OSCC) is the most common histological type of oral cancer, with high morbidity and mortality, accounting for about 90% of oral cancer [1]. Important causes of oral squamous cell carcinoma include smoking, drinking and betel nut chewing [2, 3]. It is worth mentioning that in recent years, human papillomavirus has also been found to be one of the important causes of OSCC [4, 5], which has attracted widespread attention. In the early stages, OSCC are commonly asymptomatic, it is often at advanced-stage at the time of diagnosis. Early detection of precancerous lesions in OSCC can greatly improve the survival rate of patients [6]. In addition, the low survival rate of OSCC is also related to the fact that older patients have more complication, which make them more likely to be infected with other diseases and prematurely die. In addition, there are more adverse drug reactions due to aging organ functions than younger patients. Hence, it has been suggested that different treatment strategies should be given to the young and the old [7]. In this paper, we made a survival analysis based on information of patients with oral squamous cell carcinoma in different ages.

The acquisition of the real data of large number of patients has been one of the main problems faced by researchers in the research process. We used The Surveillance, Epidemiology, and End Results (SEER) national cancer database, a cancer registry maintained by the American College of surgeons and the American Cancer Society. It records the incidence rate, mortality and morbidity of millions of malignant tumors in some states and counties in the United States. The tumor information in the database was unified and standardized by SEER*stat software, and is regularly updated and released. Researchers all over the world can easily get data through application, which provides a good data source for clinical researchers. In addition, SEER database has a large sample size cover about 34.6% of the U.S. population and strong statistical efficiency, which promoted the high clinical reference value of researches based on SEER database.

## Methods

### Data source

We collected the clinicopathological data of all 33,619 adult patients (≥ 18 years) with primary oral squamous cell carcinoma from the years of 2004 to 2015 from the SEER database. The histological type codes for squamous cell carcinoma are 8070, 8071, 8072, 8073, 8074, 8075, and 8076, according to the third edition of the *International Classification of Diseases for Oncology* (ICD-O-3). Specific information includes age, gender, race, tumor location, grade, marital status, surgery, radiation, chemotherapy, T-level, N-stage and M-stage.

### Data processing

**Fig. 1:**
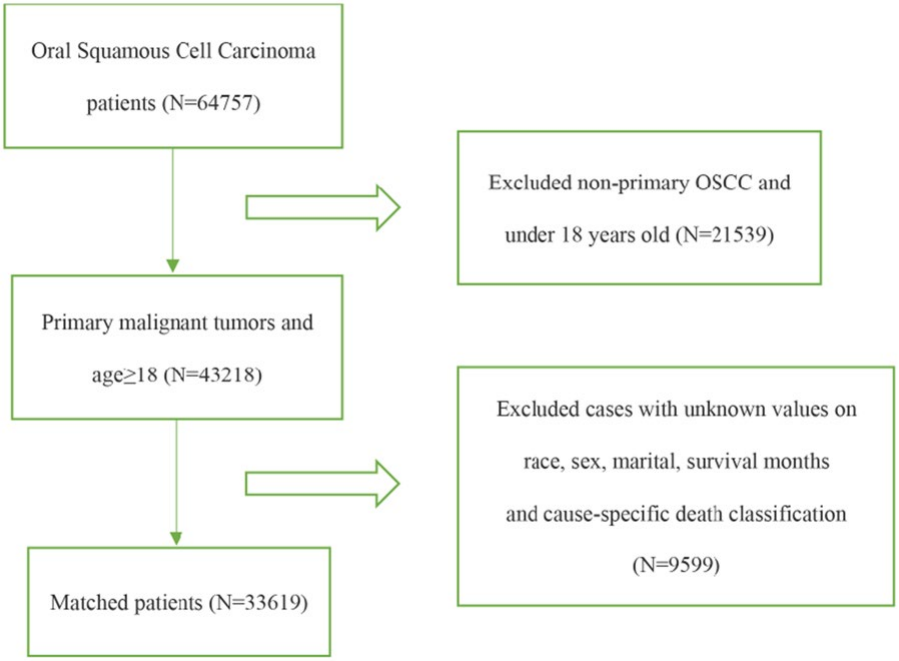
Flow chart of OSCC patient data processing

The detailed steps are explained in Fig. 1. “Age”, as the main predictor, was clustered into five groups: 18–39, 40–49, 50–59, 60–69, 70 + . The SEER “stage” was used for tumor staging according to the seventh edition of the American Joint Committee on Cancer (AJCC) manual. In this study, the primary and secondary endpoints were overall survival and cancer-specific survival, which were analyzed based on the time of diagnosis, “status” and “cause-specific death classification”. In addition, we used the ICD-O-3 code to classify the site of oral tumors: floor of mouth (C04.0–4.1, C04.8–4.9), tongue (C01.9–2.4, C02.8–2.9), other (C03.0–3.1, C03.9, C05.0–5.2, C05.8–6.2, C06.8–6.9, C07.9–8.1, C08.9–9.1, C09.8–9.9). Moreover, the treatment methods were divided into the following groups: surgery, radiation, chemotherapy + XRT, surgery + XRT, others, triple therapy, no/unknown. Other variables available for statistical analysis were also standardized in the light of the definition of the SEER database.

### Statistical analysis

All analyses were conducted in R-studio (version 4.0.2, https://www.r-proje ct.org/). We used the Kaplan–Meier method and log-rank test to obtain the Kaplan–Meier curves of 5-year overall survival rates and 5-year cancer-specific survival rates for each age, as well as Kaplan–Meier curves by age for each stage. To understand the relationship between other acquired factors and mortality of OSCC, univariate and multivariate Cox regression analyses were performed. The variables with *p* < 0.05 in univariate analysis were further analyzed by multivariate analysis.

## Results

### Patient recruitment and characteristics

A total of 33,619 cases in the SEER database were included in this study. The baseline characteristics of patients with OSCC are presented in Table 1. There were five groups stratified by age at diagnosis (18–39 years, 40–49 years, 50–59 years, 60–69 years, 70 + years). The incidence of OSCC was highest among 50–59 years (10,903, 13.24%), whereas the youngest group (18–39 years) had the least sample size. In addition, the proportion of male is much higher than that of women, especially in the group younger than 70 years. The most common site of cancer in the 18–39-year group was the tongue, which was different from other groups. The median follow‐up time of the whole cases and each group was 34 months, interquartile range 15–73 months (total), 52 months, interquartile range 19–96 months (18–39 years), 50 months, interquartile range 20–93 months (40–49 years), 40 months, interquartile range 17–79 months (50–59 years), 34 months, interquartile range 15–68 months (60–69 years), 22 months, interquartile range 9–51 months (70 + years), respectively.

**Table 1:** Clinicopathological characters of patients with oral cavity squamous cell carcinoma by age at diagnosis

Characteristics	Total	Age at diagnosis, *n* (%)				
		18–39	40–49	50–59	60–69	70 +
Total	33,619	1120 (3.33)	4408 (13.11)	10,903 (32.43)	9380 (27.90)	7808 (23.22)
Sex						
Female	9113 (27.11)	431 (38.48)	998 (22.64)	2228 (20.43)	2178 (23.22)	3278 (41.98)
Male	24,506 (72.89)	689 (61.52)	3410 (77.36)	8675 (79.57)	7202 (76.78)	4530 (58.02)
Marital status						
Married	19,106 (56.83)	560 (50.00)	2553 (57.92)	6316 (57.93)	5590 (59.59)	4087 (52.34)
Unmarried	14,513 (43.17)	560 (50.00)	1855 (42.08)	4587 (42.07)	3790 (40.41)	3721 (47.66)
Race						
American Indian/Alaska Native	185 (0.55)	3 (0.27)	32 (0.73)	70 (0.64)	51 (0.54)	29 (0.37)
Asian or Pacific Islander	1796 (5.34)	132 (11.79)	256 (5.81)	430 (3.94)	463 (4.94)	515 (6.60)
Black	2748 (8.17)	76 (6.79)	459 (10.41)	1040 (9.54)	755 (8.05)	418 (5.35)
White	28,890 (85.93)	909 (81.16)	3661 (16.95)	9363 (85.88)	8111 (86.47)	6846 (87.68)
Year of diagnosis						
2004–2010	16,818 (50.03)	637 (56.88)	2548 (57.80)	5476 (50.22)	4279 (45.62)	3860 (49.44)
2011–2015	16,801 (49.97)	483 (43.13)	1860 (42.20)	5427 (49.78)	5101 (54.38)	3948 (50.56)
Location						
Tongue	15,300 (45.51)	776 (69.29)	1902 (43.15)	4775 (43.80)	4460 (47.55)	3387 (43.38)
Floor of mouth	2433 (7.24)	25 (2.23)	290 (6.58)	843 (7.73)	729 (7.77)	546 (6.99)
Other mouth	15,886 (47.25)	319 (28.48)	2216 (50.27)	5285 (51.53)	4191 (55.32)	3875 (49.63)
Size						
< 1	2442 (7.26)	128 (11.43)	352 (7.99)	726 (6.66)	658 (7.01)	578 (7.40)
< 2	6139 (18.26)	271 (24.20)	931 (21.12)	1941 (17.80)	1666 (17.76)	1330 (17.03)
< 3	7045 (20.96)	215 (19.20)	877 (19.90)	2303 (21.12)	1932 (20.60)	1718 (22.00)
< 4	6228 (18.53)	172 (15.36)	766 (17.38)	2009 (18.43)	1812 (19.32)	1469 (18.81)
< 5	4048 (12.04)	114 (10.18)	501 (11.37)	1305 (11.97)	1140 (12.15)	988 (12.65)
≥ 5	3718 (11.06)	125 (11.16)	424 (9.62)	1166 (10.69)	1057 (11.27)	946 (12.12)
Unknown	3999 (11.90)	95 (8.48)	557 (12.64)	1453 (13.33)	1115 (11.89)	779 (9.98)
Treatment modalities						
Surgery	8100 (24.09)	382 (34.11)	923 (20.94)	2020 (18.53)	2087 (22.25)	2688 (34.43)
Radiation	1968 (5.85)	17 (1.52)	144 (3.27)	484 (4.44)	524 (5.59)	799 (10.23)
Chemotherapy + XRT	9304 (27.67)	138 (12.32)	1241 (28.15)	3497 (32.07)	3008 (32.07	1420 (18.19)
Surgery + XRT	5154 (15.33)	183 (16.34)	645 (14.63)	1582 (14.51)	1394 (14.86)	1350 (17.29)
Others	937 (2.79)	21 (1.88)	132 (2.99)	328 (3.01)	276 (2.94)	180 (2.31)
Triple therapy	6619 (19.69)	359 (32.05)	1208 (27.40)	2625 (24.08)	1684 (17.95)	743 (9.52)
No/Unknown	1537 (4.57)	20 (1.79)	115 (2.61)	367 (3.37)	407 (4.34)	628 (8.04)
Grade						
Well-differentiated	4423 (13.16)	188 (16.79)	491 (11.14)	1111 (10.19)	1151 (12.27)	1482 (18.98)
Moderately differentiated	16,467 (48.98)	593 (52.95)	2256 (51.18)	5339 (48.97)	4429 (47.22)	3850 (49.31)
Poorly differentiated	12,445 (37.02)	329 (29.38)	1618 (36.71)	4353 (39.92)	3720 (39.66)	2425 (31.06)
Undifferentiated	284 (0.84)	10 (0.89)	43 (0.98)	100 (0.92)	80 (0.85)	51 (0.65)
T classification						
T1	9795 (29.14)	431 (38.48)	1421 (32.24)	3108 (28.51)	2659 (28.35)	2176 (27.87)
T2	10,431 (31.03)	334 (29.82)	1342 (30.44)	3430 (31.46)	2951 (31.46)	2374 (30.40)
T3	4377 (13.02)	129 (11.52)	512 (11.62)	1371 (12.57)	1262 (13.45)	1103 (14.13)
T4	6294 (18.72)	155 (13.84)	754 (17.11)	2001 (18.35)	1776 (18.93)	1608 (20.59)
Unknown	2722 (8.10)	71 (6.34)	379 (8.60)	993 (9.11)	732 (7.80)	547 (7.01)
N classification						
N0	12,381 (36.83)	518 (46.25)	1360 (30.85)	3250 (29.81)	3208 (34.20)	4045 (51.81)
N1	6022 (17.91)	179 (15.98)	784 (17.79)	1999 (18.33)	1744 (18.59)	1316 (16.85)
N2	13,683 (40.70)	383 (34.20)	2056 (46.64)	5096 (46.74)	3983 (42.46)	2165 (27.73)
N3	1135 (3.38)	28 (2.50)	166 (3.77)	466 (4.27)	340 (3.62)	135 (1.73)
Unknown	398 (1.18)	12 (1.07)	42 (0.95)	92 (0.84)	105 (1.12)	147 (1.88)
M classification						
M0	32,461 (96.56)	1100 (98.21)	4283 (97.16)	10,540 (96.67)	9027 (96.24)	7511 (96.20)
M1	988 (2.94)	13 (1.16)	100 (2.27)	310 (2.84)	313 (3.34)	252 (3.23)
Unknown	170 (0.51)	7 (0.63)	25 (0.57)	53 (0.49)	40 (0.43)	45 (0.58)
Stage						
1	5339 (15.88)	283 (25.27)	657 (14.90)	1440 (13.21)	1377 (14.68)	1582 (20.26)
2	3548 (10.55)	136 (12.14)	384 (8.71)	937 (8.59)	924 (9.85)	1167 (14.95)
3	5612 (16.69)	193 (17.23)	708 (16.06)	1754 (16.09)	1597 (17.03)	1360 (17.42)
4	17,993 (53.52)	475 (42.41)	2543 (57.69)	6449 (59.15)	5156 (54.97)	3370 (43.16)
Unknown	1127 (3.35)	33 (2.95)	116 (2.63)	323 (2.96)	326 (3.48)	329 (4.21)
Status						
Alive	19,464 (57.90)	824 (73.57)	3033 (68.81)	7129 (65.39)	5553 (59.20)	2925 (37.46)
Dead (attributable to cancer)	10,623 (31.60)	275 (24.55)	1129 (25.61)	3019 (27.69)	2920 (31.13)	3280 (42.01)
Dead of other cause	3532 (10.51)	21 (1.88)	246 (5.58)	755 (6.92)	907 (9.67)	1603 (20.53)
Follow‐up time	34 (15, 73)	52 (19, 96)	50 (20, 93)	40 (17, 79)	34 (15, 68)	22 (9, 51)

### Survival analyses of OSCC according to age at diagnosis

**Fig. 2:**
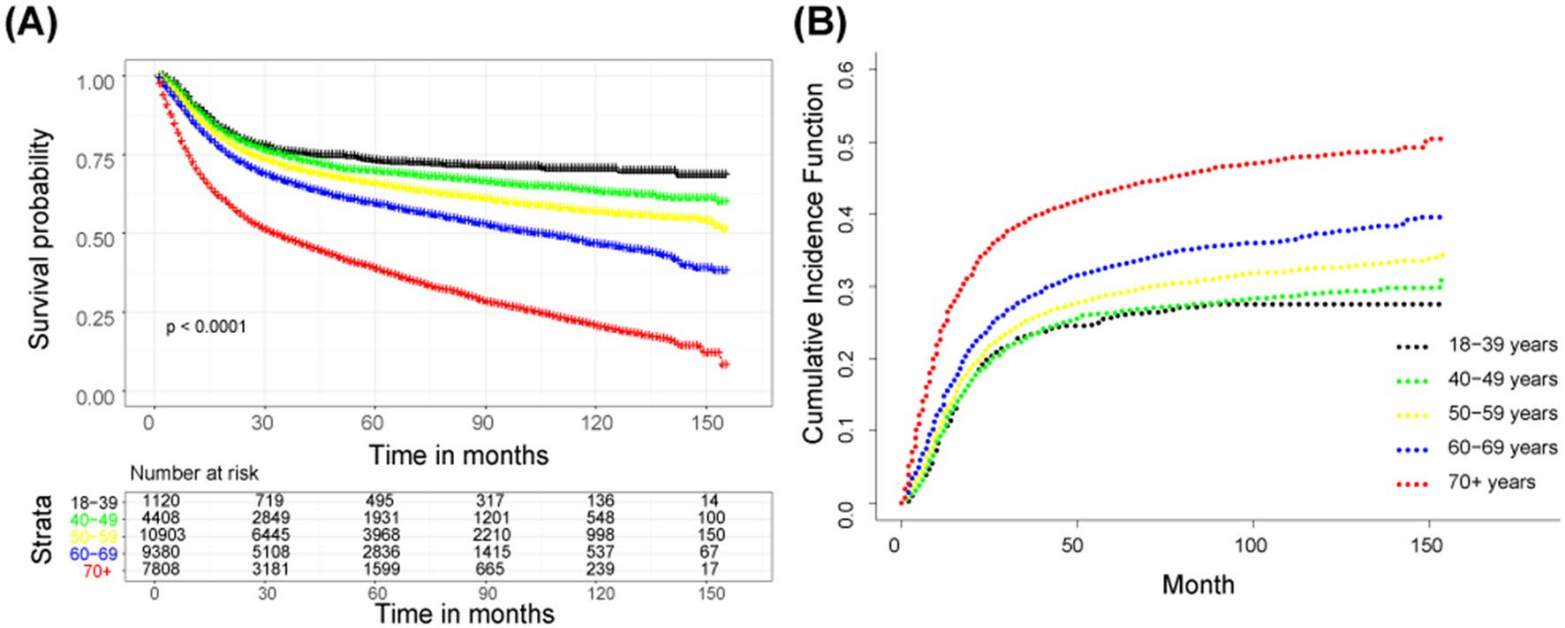
Kaplan–Meier survival curves and cumulative incidence function for 5-year OS/CSS. **A** Survival curve of patients with OSCC at different ages; **B** cumulative incidence function of patients with OSCC at different ages

Figure 2A presents the 5‐year OS for OSCC decreased with age analyzed by Kaplan–Meier. With the extension of the follow-up time, the differences between the groups were larger. The oral squamous cell cancer-specific survival among 18–39-year, 40–49-year, 50–59-year, 60–69-year, 70 +-year group also gave a similar result (Fig. 2B), revealing that age had a major influence on survival time.

**Fig. 3:**
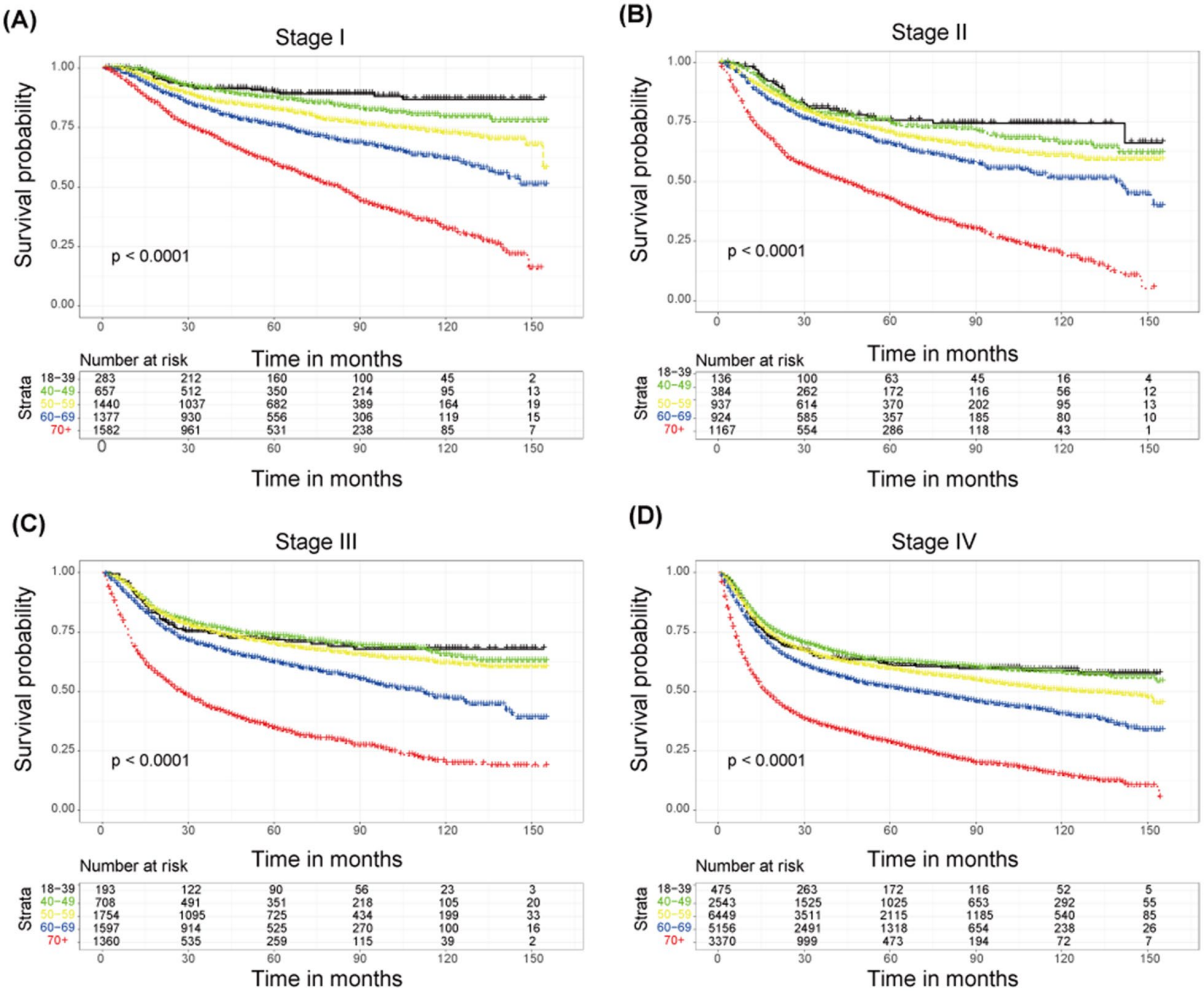
Kaplan–Meier survival curves grouped by age at each stage. **A** Survival curve of stage I OSCC patients at different ages; **B** survival curve of stage II OSCC patients at different ages; **C** survival curve of stage III OSCC patients at different ages; **D** survival curve of stage IV OSCC patients at different ages

**Fig. 4:**
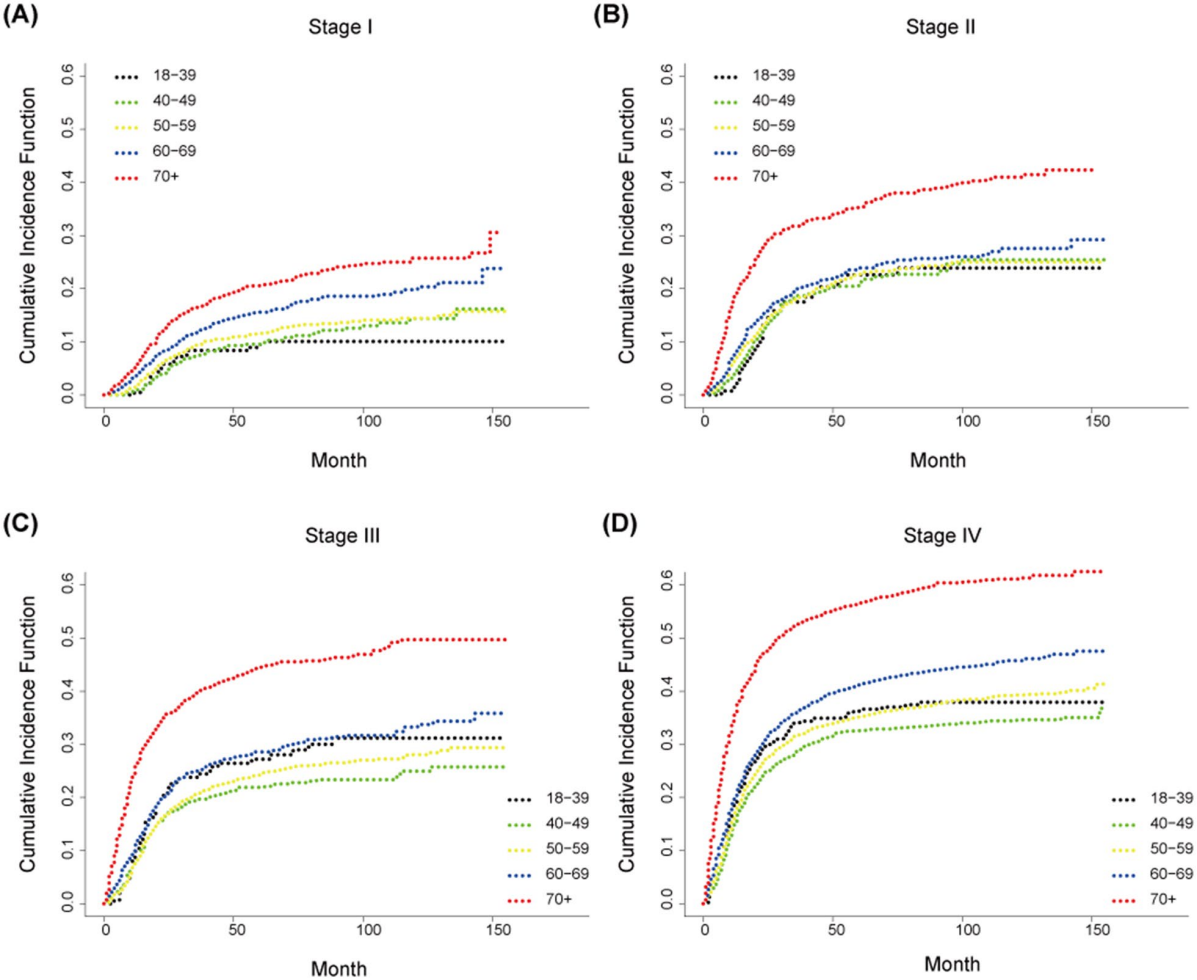
Cumulative incidence function grouped by age at each stage. **A** Cumulative incidence function of stage I OSCC patients at different ages; **B** cumulative incidence function of stage II OSCC patients at different ages; **C** cumulative incidence function of stage III OSCC patients at different ages; **D** cumulative incidence function of stage IV OSCC patients at different ages

Kaplan–Meier survival curves as well as cumulative incidence function divided by age at each stage are produced (Figs. 3 and 4). The 5-year OS of stages I–II were similar to that of the general population, but for stages III and IV, only those aged over 60 had significant difference in survival rate, while the three groups of 18–39 years, 40–49 years and 50–59 years were similar, which indicated that age was an important factor in explaining the difference of survival, but not the only factor (Fig. 3). As for the result of CSS in different stages, the elderly group, especially 70 +-year and 60–69-year patients, still have a significant difference connection with cancer‐specific death (Fig. 4).

When we conducted univariate and multivariate analyses targeting overall survival (OS) and cancer‐specific survival (CSS), as expected, age, sex, marital status, race, tumor location and size, treatment, pathological grade and TNM staging were covariates in the adjusted model, which showed statistical significance (*P* < 0.05) (Tables 2, and 3). Older age (≥ 50 years) was an important predictor of worse prognosis at all stages compared with patients aged 18–39. The specific value was 50–59 years (HR, 1.32; 95% CI 1.17–1.48; *p* ≤ 0.001), 60–69 years (HR, 1.66; 95% CI 1.42–1.87; *p* ≤ 0.001) and 70 + years (HR, 3.21; 95% CI 2.86–3.62; *p* ≤ 0.001). While the competing risk model was 60–69 years (HR, 1.21; 95% CI 1.07–1.38; *p* = 0.002) and 70 + years (HR, 1.85; 95% CI 1.63–2.10; *p* ≤ 0.001). In addition, Tables 2 and 3 also reveal other predictors that signify significant clinically deterioration of OS/CSS in univariate and multivariate regression analyses included female gender, unmarried, Blacks, tumor in floor of mouth, size and higher TNM classification.

**Table 2:** Univariate and multivariable cox regression analyses of OS in oral cavity squamous cell carcinoma

Clinicopathological variables	Univariate analysis		Multivariate analysis	
	HR (95%CI)	*P*	HR (95%CI)	*P*
Age at diagnosis				
18–39	Reference		Reference	
40–49	1.19 (1.05–1.35)	0.007	1.15 (1.01–1.30)	0.034
50–59	1.43 (1.27–1.61)	< 0.001	1.32 (1.17–1.48)	< 0.001
60–69	1.83 (1.62–2.06)	< 0.001	1.66 (1.42–1.87)	< 0.001
70 +	3.46 (3.07–3.89)	< 0.001	3.21 (2.86–3.62)	< 0.001
Sex				
Female	Reference		Reference	
Male	0.83 (0.80–0.86)	< 0.001	0.94 (0.90–0.97)	< 0.001
Marital status				
Married	Reference		Reference	
Unmarried	1.84 (1.78–1.90)	< 0.001	1.48 (1.43–1.54)	< 0.001
Race				
White	Reference		Reference	
Black	1.82 (1.73–1.92)	< 0.001	1.34 (1.27–1.41)	< 0.001
Asian or Pacific Islander	0.96 (0.89–1.04)	0.340	1.02 (0.95–1.11)	0.566
American Indian/Alaska Native	1.08 (0.86–1.36)	0.482	1.04 (0.83–1.31)	0.743
Location				
Floor of mouth	Reference		Reference	
Other mouth	0.62 (0.58–0.66)	< 0.001	0.60 (0.56–0.63)	< 0.001
Tongue	0.65 (0.62–0.69)	< 0.001	0.71 (0.67–0.76)	< 0.001
Size				
< 1	Reference		Reference	
< 2	1.38 (1.25–1.53)	< 0.001	1.35 (1.22–1.49)	< 0.001
< 3	1.96 (1.79–2.16)	< 0.001	1.63 (1.45–1.82)	< 0.001
< 4	2.49 (2.26–2.73)	< 0.001	1.91 (1.69–2.16)	< 0.001
< 5	3.36 (3.05–3.70)	< 0.001	2.29 (2.02–2.60)	< 0.001
≥ 5	5.48 (4.98–6.03)	< 0.001	3.11 (2.74–3.54)	< 0.001
Unknown	3.17 (2.88–3.50)	< 0.001	2.40 (2.09–2.75)	< 0.001
Treatment				
No/Unknown	Reference		Reference	
Surgery	0.18 (0.18–0.19)	< 0.001	0.30 (0.28–0.32)	< 0.001
Radiation	0.44 (0.41–0.48)	< 0.001	0.48 (0.44–0.52)	< 0.001
Chemotherapy + XRT	0.23 (0.22–0.25)	< 0.001	0.26 (0.24–0.27)	< 0.001
Surgery + XRT	0.21 (0.19–0.22)	< 0.001	0.28 (0.26–0.30)	< 0.001
Triple therapy	0.18 (0.17–0.19)	< 0.001	0.25 (0.23–0.27)	< 0.001
Others	0.63 (0.58–0.70)	< 0.001	0.54 (0.49–0.60)	< 0.001
Grade				
Well-differentiated	Reference		Reference	
Moderately differentiated	1.15 (1.09–1.21)	< 0.001	1.07 (1.02–1.13)	0.009
Poorly differentiated	0.93 (0.88–0.98)	0.006	0.86 (0.81–0.91)	< 0.001
Undifferentiated	0.83 (0.68–1.02)	0.079	0.78 (0.63–0.95)	0.015
T classification				
T1	Reference		Reference	
T2	1.61 (1.53–1.69)	< 0.001	1.13 (1.04–1.22)	0.004
T3	2.67 (2.52–2.82)	< 0.001	1.24 (1.13–1.37)	< 0.001
T4	3.84 (3.66–4.04)	< 0.001	1.89 (1.73–2.06)	< 0.001
Unknown	1.85 (1.72–1.98)	< 0.001	0.82 (0.73–0.92)	< 0.001
N classification				
N0	Reference		Reference	
N1	1.25 (1.19–1.31)	< 0.001	1.36 (1.29–1.43)	< 0.001
N2	1.25 (1.20–1.30)	< 0.001	1.38 (1.32–1.45)	< 0.001
N3	1.71 (1.58–1.86)	< 0.001	1.75 (1.60–1.92)	< 0.001
Unknown	2.64 (2.33–3.00)	< 0.001	1.44 (1.26–1.65)	< 0.001
M classification				
M0	Reference		Reference	
M1	4.00 (3.72–4.29)	< 0.001	2.54 (2.36–2.74)	< 0.001
Unknown	2.50 (2.11–2.97)	< 0.001	1.38 (1.16–1.65)	< 0.001

**Table 3:** Univariate and multivariable competing risk model regression analyses of CSS in oral cavity squamous cell carcinoma

Clinicopathological variables	Univariate analysis		Multivariate analysis	
	SHR (95%CI)	*P*	SHR (95%CI)	*P*
Age at diagnosis				
18–39	Reference		Reference	
40–49	1.04 (0.91–1.18)	0.590	0.98 (0.86–1.12)	0.800
50–59	1.17 (1.04–1.32)	0.011	1.06 (0.94–1.20)	0.360
60–69	1.37 (1.21–1.55)	< 0.001	1.21 (1.07–1.38)	0.002
70 +	2.05 (1.82–2.32)	< 0.001	1.85 (1.63–2.10)	< 0.001
Sex				
Female	Reference		Reference	
Male	0.84 (0.81–0.88)	< 0.001	0.85 (0.81–0.89)	< 0.001
Marital status				
Married	Reference		Reference	
Unmarried	1.71 (1.65–1.78)	< 0.001	1.33 (1.27–1.38)	< 0.001
Race				
White	Reference		Reference	
Black	1.86 (1.76–1.97)	< 0.001	1.29 (1.20–1.38)	< 0.001
Asian or Pacific Islander	1.08 (0.99–1.18)	0.072	1.18 (1.08–1.29)	< 0.001
American Indian/Alaska Native	1.11 (0.86–1.42)	0.430	1.06 (0.82–1.36)	0.650
Location				
Floor of mouth	Reference		Reference	
Other mouth	0.64 (0.60–0.69)	< 0.001	0.60 (0.55–0.64)	< 0.001
Tongue	0.72 (0.68–0.77)	< 0.001	0.76 (0.71–0.82)	< 0.001
Size				
< 1	Reference		Reference	
< 2	1.65 (1.45–1.88)	< 0.001	1.51 (1.33–1.72)	< 0.001
< 3	2.49 (2.20–2.82)	< 0.001	1.83 (1.57–2.12)	< 0.001
< 4	3.21 (2.84–3.64)	< 0.001	2.11 (1.80–2.47)	< 0.001
< 5	4.56 (4.02–5.17)	< 0.001	2.59 (2.20–3.05)	< 0.001
≥ 5	7.37 (6.51–8.34)	< 0.001	3.39 (2.87–4.00)	< 0.001
Unknown	4.46 (3.94–5.06)	< 0.001	2.73 (2.29–3.25)	< 0.001
Treatment				
No/unknown	Reference		Reference	
Surgery	0.163 (0.15–0.18)	< 0.001	0.33 (0.29–0.37)	< 0.001
Radiation	0.441 (0.40–0.49)	< 0.001	0.53 (0.47–0.59)	< 0.001
Chemotherapy + XRT	0.28 (0.26–0.30)	< 0.001	0.33 (0.30–0.36)	< 0.001
Surgery + XRT	0.23 (0.21–0.25)	< 0.001	0.35 (0.32–0.39)	< 0.001
Triple therapy	0.23 (0.21–0.25)	< 0.001	0.34 (0.31–0.38)	< 0.001
Others	0.74 (0.66–0.83)	< 0.001	0.65 (0.57–0.75)	< 0.001
Grade				
Well-differentiated	Reference		Reference	
Moderately differentiated	1.28 (1.21–1.36)	< 0.001	1.08 (1.01–1.16)	0.023
Poorly differentiated	1.05 (0.99–1.12)	0.130	0.86 (0.80–0.93)	< 0.001
Undifferentiated	0.87 (0.69–1.11)	0.260	0.69 (0.54–0.88)	0.003
T classification				
T1	Reference		Reference	
T2	1.80 (1.70–1.92)	< 0.001	1.17 (1.06–1.30)	0.002
T3	3.09 (2.89–3.30)	< 0.001	1.29 (1.15–1.45)	< 0.001
T4	4.48 (4.23–4.75)	< 0.001	1.94 (1.74–2.16)	< 0.001
Unknown	2.18 (2.01–2.36)	< 0.001	0.85 (0.73–0.98)	0.025
N classification				
N0	Reference		Reference	
N1	1.52 (1.43–1.60)	< 0.001	1.48 (1.39–1.58)	< 0.001
N2	1.63 (1.55–1.70)	< 0.001	1.56 (1.47–1.65)	< 0.001
N3	2.34 (2.14–2.57)	< 0.001	2.06 (1.84–2.31)	< 0.001
Unknown	2.87 (2.46–3.35)	< 0.001	1.50 (1.26–1.79)	< 0.001
M classification				
M0	Reference		Reference	
M1	4.39 (4.05–4.76)	< 0.001	2.62 (2.36–2.90)	< 0.001
Unknown	2.60 (2.11–3.22)	< 0.001	1.47 (1.12–1.93)	0.006

## Discussion

Age has always been an important factor in the occurrence, development and prognosis of various tumor. Squamous cell carcinoma of the head and neck (HNSCC) is generally considered to be more frequent in the elderly, associated with tobacco and alcohol, and mainly occurs in men [8]. However, more and more young patients with HNSCC have been reported all over the world [9]. For the past few years, the incidence of OSCC has been on the rise, especially among young patients [10].The purpose of this SEER database analysis was to assess the clinical characteristics and risk factors of OSCC in different age groups. At the same time, understanding of other factors (gender, tumor size, histological grade, treatment, etc.) that affect the premature death of patients will help to formulate the corresponding treatment plan in advance and improve the survival rate. To our knowledge, this study is the first to observe the possible differences stratified by age in studies with a large sample size.

As we expected, whether it is OS or CSS, the research shows that the survival time of patients decreases orderly and stepwise with the increase of age group. This result is consistent with other large cohort studies that have been published. A study carried out in Brazil shown that age has a strong impact on mortality from oral and oropharyngeal cancer. The risk increases from 40 years for men to 55 years for women and the effect of the overall period was observed [11]. Laith et al. reported that their study indicated improved OS and disease-specific survival in young patients with oral tongue squamous cell carcinoma (OTSCC) [12]. However, another interesting finding of the regression analysis is that compared with the higher age group, people aged < 30 showed a higher probability of transition, which is not statistically significant [13]. Younger age at diagnosis even was found to be a risk factor for the development of pleural metastasis [14]. In general, the effect of age on the prognosis of OSCC is still controversial. Although a number of studies have made different results, they are unable to explain the etiology and pathological mechanism in detail. From our analysis of the results, young patients (18–39) had a higher rate of surgery (34.11%) and triple therapy (32.05%), indicating that they tend to accept more aggressive treatments.

It has reported that 5-year survival rates for patients with oral squamous cell carcinoma vary greatly by stage, from about 90% in the early stage to about 30% in the late stage [15]. Surgery is the main treatment for early (Stages I–II) oral squamous cell carcinoma. Advanced (Stages III–IV) disease indicates difficulty in obtaining a clear incision margin, which means a higher recurrence rate. Under the circumstance, adjuvant therapy is appropriate [16]. Our research found that age has different effects on prognosis at different stages. In the early stage, the patient's survival period decreased with increasing age. As the stage progresses, the impact of different age groups on the prognosis is less obvious, which is mainly reflected in the poor prognosis of the elderly. Therefore, clinical staging at diagnosis is important and can be used as a predictor of recurrence and death in patients with OSCC.

Based on the results of previous studies, the most common major sites involved in OSCC vary by geographic location. The buccal mucosa is more common in Asian populations, including South Asia, Sri Lanka, etc., where 40% of oral cancers are found in the buccal mucosa due to the common practice of men and women chewing betel nut/tobacco. In contrast, the tongue is the most common site of oral cancer in European and American populations, accounting for 40–50% of oral cancers [17, 18]. The main source of cases in our study is mostly white Americans and our results for the location of OSCC are also within this range. It is worth noting that the proportion of tongue cancer patients is the highest in the 18–39-year-old group (69.29%). This is consistent with a previous study based on a global database analysis [19]. However, the incidence factors of young people are still unclear, and may be related to changes in the etiology of oral cancer, such as human papilloma virus (HPV) infection. In addition, the Centers for Disease Control and Prevention (CDC) and the Food and Drug Administration (FDA) analyzed data from the 2011–2015 National Youth Tobacco Survey (NYTS) and determined that the use of e-cigarettes and hooks by middle school students has increased significantly, and the trend is much larger than that of adults [20]. However, it is still necessary to further investigate the influence of young people’s eating habits, lifestyle and other factors on their incidence and tumor location.

As a retrospective study, we acknowledge that there are certain limitations to the study. As for SEER database, a large population retrospective database, inevitably, it has some drawbacks. It does not provide the data of detailed immunohistochemical analysis, for example. It also lacks related chemotherapy or radiotherapy regimens. However, the strengths of our study include a large nationally representative sample, meticulous grouping of age, as well as a wealth of other relevant factors.

## Conclusions

Our study revealed that age was an independent predictor of both OS and CSS in the oral squamous cell carcinoma patients, and more aggressive treatments (surgery, triple therapy) tend to be used in young patients, which can provide certain reference value for the current clinical diagnosis and treatment.

## Data Availability

The data sets generated and/or analyzed during the current study are freely available in the SEER repository, [https://seer.cancer.gov/seerstat/].

## Abbreviations

OSCC: Oral squamous cell carcinoma
OS: Overall survival
CSS: Cancer-specific survival
SEER: The Surveillance, Epidemiology, and End Results
TNM: Tumor node metastasis
CI: Confidence interval
HR: Hazard ratios
AJCC: American Joint Committee on Cancer
XRT: X-ray diffraction topography
HNSCC: Squamous cell carcinoma of the head and neck
HPV: Human papilloma virus
CDC: Centers for Disease Control and Prevention
FDA: Food and Drug Administration
NYTS: National Youth Tobacco Survey
